# Medium to Long Range Kinematic GPS Positioning with Position-Velocity-Acceleration Model Using Multiple Reference Stations

**DOI:** 10.3390/s150716895

**Published:** 2015-07-13

**Authors:** Chang-Ki Hong, Chi Ho Park, Joong-hee Han, Jay Hyoun Kwon

**Affiliations:** 1 Department of Geoinformatics Engineering, Kyungil University, 50 Gamasilgil, Kyeongsan, Gyeongbuk 712-701, Korea; E-Mail: ckhong@kiu.ac.kr; 2 Division of IoT and Robotics Convergence Research, Daegu Gyeongbuk Institute of Science & Technology, 333 Techno jungang-daero, Hyeonpung-myeon, Dalseong-gun, Dagegu 711-873, Korea; E-Mail: chpark@dgist.ac.kr; 3 Department of Geoinformatics, University of Seoul, 163 Seoulsiripdaero, Dongdaemun-gu, Seoul 130-743, Korea; E-Mail: hjh0016@uos.ac.kr

**Keywords:** global positioning system (GPS), kinematic acceleration, position-velocity-acceleration model

## Abstract

In order to obtain precise kinematic global positioning systems (GPS) in medium to large scale networks, the atmospheric effects from tropospheric and ionospheric delays need to be properly modeled and estimated. It is also preferable to use multiple reference stations to improve the reliability of the solutions. In this study, GPS kinematic positioning algorithms are developed for the medium to large-scale network based on the position-velocity-acceleration model. Hence, the algorithm can perform even in cases where the near-constant velocity assumption does not hold. In addition, the estimated kinematic accelerations can be used for the airborne gravimetry. The proposed algorithms are implemented using Kalman filter and are applied to the *in situ* airborne GPS data. The performance of the proposed algorithms is validated by analyzing and comparing the results with those from reference values. The results show that reliable and comparable solutions in both position and kinematic acceleration levels can be obtained using the proposed algorithms.

## 1. Introduction

For the past two decades, global positioning systems (GPS) has been widely used for precise positioning in various engineering and scientific fields. While its application areas in the early stage were limited to the determination of a position at a static environment, more and more applications which require kinematic positioning have been developed. For the kinematic GPS positioning, either single or multi-reference stations can be used for the data processing. The multi-reference station approach generally offers better positioning accuracy and reliability [1,2]. Therefore, the multi-reference station approach is recommended when precise kinematic positioning is required. However, many distance-dependent errors, such as atmospheric and ionospheric delays, are not fully removed through the double-differencing technique when the baseline length increases. Thus, these errors should be properly modeled for the medium to long range kinematic positioning [3,4,5]. GPS kinematic positioning is usually performed by using the Kalman filter, and so called Position-Velocity (PV) model can be adopted when the object is assumed to move with a nearly constant velocity. In addition, Position-Velocity-Acceleration (PVA) model can be introduced for the cases where the near-constant velocity assumption does not hold [6]. This means that the PVA model can provide not only the flexibility in estimation procedure but also the position, velocity, and kinematic acceleration of the moving object simultaneously.

It is notable that one of the popular applications utilizing the GPS derived acceleration is the airborne gravimetry. The airborne gravity survey is performed by measuring the kinematic accelerations of the aircraft using GPS. Then, the gravity information is extracted using the well-known equation as shown in Equation (1). The Equation (1) expressed in inertial frame shows that the kinematic accelerations consist of the gravitational acceleration and the specific force sensed by an accelerometer [7,8];

(1) $$\ddot{x}=g+a$$

where $\ddot{x}$ is kinematic acceleration; g is gravitational acceleration; a is specific force.

In general, the GPS measurements are processed in relative positioning mode to obtain accurate and reliable positioning results in 3-D space. Then, the kinematic accelerations are computed by taking second-order time-derivatives of positions [9,10]. This means that two steps of data processing are required to get the kinematic accelerations from the positions, which decrease the efficiency in a computational point of view. To overcome the drawback of this method, one can also use the PVA model approach so that the position, velocity, and acceleration of the aircraft can be determined at the same time. An efficient PVA model for Kalman filter is proposed and demonstrated using the measurements from the electronic tacheometer, *i.e.*, geodetic kinematic measurements [11]. However, it should be noted that the experiments are conducted in an indoor environment, and the measurement used in the experiments are not from the GPS.

In this paper, the PVA model to directly determine not only the positions but also the kinematic accelerations is proposed, and the validation of the algorithm is demonstrated with *in situ* GPS measurements. The proposed algorithm is designed for the medium to long baseline scenario so that it can perform properly even in a survey for a large area under unfavorable surveying condition. The atmospheric effects such as tropospheric and ionospheric delays are modeled in the Kalman filter for the medium to long baseline scenario. In addition, multi-baseline approach is adopted for the improvement of the accuracy and reliability of the solutions. The algorithms are developed and numerical test are performed to validate the proposed algorithms by analyzing the positioning results and comparing the kinematic accelerations with those from the second-order time-derivatives of positions.

## 2. Methodology

The Kalman filter is an optimal recursive estimator which uses a system’s dynamics model and sequential measurements to estimate the system’s unknown states in a minimum variance sense. The Equation (2) shows the discrete Kalman filter equations categorized into two components, *i.e.*, the prediction of state vector through the state-transition matrix and the update of the state vector using the measurements [12,13].

(2) $$\begin{array}{c} x_{k}=\Phi_{k-1}x_{k-1}+w_{k-1}\;\;w_{k}\sim N\left(0,Q_{k}\right)\\ z_{k}=H_{k}x_{k}+v_{k}\;\;\;\;\;v_{k}\sim N\left(0,R_{k}\right) \end{array}$$

where subscript k indicates the epoch; $x_{k}$ is state vector; $\Phi_{k}$ is state-transition matrix; $w_{k}$ is process noise vector; $Q_{k}$ is covariance matrix for the processes noise; $z_{k}$ is measurement vector; $H_{k}$ is design matrix; $v_{k}$ is measurement noise vector; $R_{k}$ is covariance matrix for the measurement noise.

### 2.1. State Vector and System Equation

The objective of the proposed algorithm is to determine the kinematic accelerations of the aircraft in unfavorable conditions. Therefore, the algorithm is designed for the medium to long baseline scenario implementing multi-baseline data processing. This means that the proper modeling for the atmospheric effects such as tropospheric and ionospheric delays is required and, as a consequence, the zenith wet delay (ZWD) residuals and the double-differenced (DD) ionospheric delays are included in the state vector as shown in Equation (3). The system state vector is composed of positions of reference stations ($x_{i\_pos}$), position-velocity-acceleration of aircraft ($x_{j\_pva}$), zenith wet delay residual (ΔZWD), ambiguities for L1 and wide-lane ($x_{amb}$), and DD ionospheric delay ($x_{ion}$) as follows:

(3) $$x_{k}={\left[\begin{array}{ccccc} \underset{x_{i\_pos}}{\underset{︸}{\begin{array}{ccc} x_{i} & y_{i} & z_{i} \end{array}}} & \underset{x_{j\_pva}}{\underset{︸}{\begin{array}{ccccccccc} x_{j} & {\dot{x}}_{j} & {\ddot{x}}_{j} & y_{j} & {\dot{y}}_{j} & {\ddot{y}}_{j} & z_{j} & {\dot{z}}_{j} & {\ddot{z}}_{j} \end{array}}} & \underset{x_{ZWD}}{\underset{︸}{\begin{array}{cc} \Delta ZWD_{i} & \Delta ZWD_{j} \end{array}}} & \underset{x_{amb}}{\underset{︸}{\begin{array}{cc} N_{ij,1}^{k\ell } & N_{ij,w}^{k\ell } \end{array}}} & \underset{x_{ion}}{\underset{︸}{I_{ij}^{k\ell }/f_{1}^{2}}} \end{array}\right]}^{T}$$

where subscript i and j denote reference station and the moving aircraft, respectively; superscript k and ℓ are the satellite indices; x, y, z are the position components; $\dot{x}$, $\dot{y}$, $\dot{z}$ and $\ddot{x}$, $\ddot{y}$, $\ddot{z}$ are velocities and accelerations of the aircraft; ΔZWD is the total zenith tropospheric delay residual; $N_{ij,1}^{k\ell }$ and $N_{ij,w}^{k\ell }\;\left(=N_{ij,1}^{k\ell }-N_{ij,2}^{k\ell }\right)$ are the DD ambiguities for L1 and wide-lane, respectively; $I_{ij}^{k\ell }/f_{1}^{2}$ is the DD ionospheric delay.

The corresponding state-transition matrix can be expressed by:

(4) $$\Phi =\left[\begin{array}{ccccc} \Phi_{i\_pos} & 0 & 0 & 0 & 0\\ 0 & \Phi_{j\_pva} & 0 & 0 & 0\\ 0 & 0 & \Phi_{\Delta ZWD} & 0 & 0\\ 0 & 0 & 0 & \Phi_{amb} & 0\\ 0 & 0 & 0 & 0 & \Phi_{ion} \end{array}\right]$$

The coordinates of reference stations are included in the state vector and estimated with an assumption of random constant stochastic process, so the transition matrix for those are given as in Equation (5).

(5) $$\Phi_{i\_pos}=\left[\begin{array}{ccc} 1 & 0 & 0\\ 0 & 1 & 0\\ 0 & 0 & 1 \end{array}\right]$$

The state-transition matrix for position-velocity-acceleration components of the aircraft can be derived with the assumption that the kinematic acceleration is Gaussian white noise process [6,11,14]:

(6) $$\Phi_{j\_pva}=\left[\begin{array}{ccccccccc} 1 & \Delta t & \frac{{\left(\Delta t\right)}^{2}}{2} & 0 & 0 & 0 & 0 & 0 & 0\\ 0 & 1 & \Delta t & 0 & 0 & 0 & 0 & 0 & 0\\ 0 & 0 & 1 & 0 & 0 & 0 & 0 & 0 & 0\\ 0 & 0 & 0 & 1 & \Delta t & \frac{{\left(\Delta t\right)}^{2}}{2} & 0 & 0 & 0\\ 0 & 0 & 0 & 0 & 1 & \Delta t & 0 & 0 & 0\\ 0 & 0 & 0 & 0 & 0 & 1 & 0 & 0 & 0\\ 0 & 0 & 0 & 0 & 0 & 0 & 1 & \Delta t & \frac{{\left(\Delta t\right)}^{2}}{2}\\ 0 & 0 & 0 & 0 & 0 & 0 & 0 & 1 & \Delta t\\ 0 & 0 & 0 & 0 & 0 & 0 & 0 & 0 & 1 \end{array}\right]$$

For the modeling of tropospheric effect, either random walk or first-order Gauss-Markov process is used because either process is known to be sufficient to describe the stochastic property of tropospheric effect [4]. Here, the zenith wet delay residual, *i.e.*, increment with respect to *a priori* value, is assumed to be random walk stochastic process and the transition matrix is:

(7) $$\Phi_{\Delta ZWD}=\left[\begin{array}{cc} 1 & 0\\ 0 & 1 \end{array}\right]$$

The remaining atmospheric effect, *i.e.*, DD ionospheric delays, is modeled with first-order Gauss-Markov process. The first-order Gauss-Markov process can describe a large number of physical processes with reasonable accuracy [15]. However, it should be noted that the DD ionospheric delays are correlated with not only time but also baseline length. Hence, the DD ionospheric delays are modeled with the first-order Gauss-Markov process which incorporates the effect of both the time and baseline length changes as shown by [3].

(8) $$\Phi_{ion}=e^{-\left(\Delta t/T+\left|\delta \right|/D\right)}$$

where *T* is first-order correlation time for the DD ionosphere; |δ| is baseline length change; D=1500 km is the first-order correlation distance.

The DD ambiguities for L1 and wide-lane measurements can be modeled as a random constant process as the case of reference stations’ coordinates. It should be noted that the ambiguity resolution is not attempted because it is an issue outside the scope of this study in long range static or kinematic positioning.

For the Kalman filtering, the process noise covariance matrix should be properly modeled as it confines the variability of the state estimates. The discrete form of covariance matrices for the reference stations’ coordinates and the position-velocity-acceleration of aircraft can be written as shown in Equations (9) and (10), respectively [6,11].

(9) $$Q_{i\_pos}=\left[\begin{array}{ccc} 0 & 0 & 0\\ 0 & 0 & 0\\ 0 & 0 & 0 \end{array}\right]$$

(10) $$Q_{j\_pva}=q_{acc}\cdot \left[\begin{array}{ccccccccc} \frac{{\left(\Delta t\right)}^{5}}{20} & \frac{{\left(\Delta t\right)}^{4}}{8} & \frac{{\left(\Delta t\right)}^{3}}{6} & 0 & 0 & 0 & 0 & 0 & 0\\ \frac{{\left(\Delta t\right)}^{4}}{8} & \frac{{\left(\Delta t\right)}^{3}}{3} & \frac{{\left(\Delta t\right)}^{2}}{2} & 0 & 0 & 0 & 0 & 0 & 0\\ \frac{{\left(\Delta t\right)}^{3}}{6} & \frac{{\left(\Delta t\right)}^{2}}{2} & \Delta t & 0 & 0 & 0 & 0 & 0 & 0\\ 0 & 0 & 0 & \frac{{\left(\Delta t\right)}^{5}}{20} & \frac{{\left(\Delta t\right)}^{4}}{8} & \frac{{\left(\Delta t\right)}^{3}}{6} & 0 & 0 & 0\\ 0 & 0 & 0 & \frac{{\left(\Delta t\right)}^{4}}{8} & \frac{{\left(\Delta t\right)}^{3}}{3} & \frac{{\left(\Delta t\right)}^{2}}{2} & 0 & 0 & 0\\ 0 & 0 & 0 & \frac{{\left(\Delta t\right)}^{3}}{6} & \frac{{\left(\Delta t\right)}^{2}}{2} & \Delta t & 0 & 0 & 0\\ 0 & 0 & 0 & 0 & 0 & 0 & \frac{{\left(\Delta t\right)}^{5}}{20} & \frac{{\left(\Delta t\right)}^{4}}{8} & \frac{{\left(\Delta t\right)}^{3}}{6}\\ 0 & 0 & 0 & 0 & 0 & 0 & \frac{{\left(\Delta t\right)}^{4}}{8} & \frac{{\left(\Delta t\right)}^{3}}{3} & \frac{{\left(\Delta t\right)}^{2}}{2}\\ 0 & 0 & 0 & 0 & 0 & 0 & \frac{{\left(\Delta t\right)}^{3}}{6} & \frac{{\left(\Delta t\right)}^{2}}{2} & \Delta t \end{array}\right]$$

The initial spectral density, $q_{acc}$ for the acceleration states can be determined using the initial kinematic solutions by computing the standard deviation of the time-differenced accelerations [14]. In this study, $q_{acc}$ is set to 1 m^2^/s^4^/Hz for the data processing through the analysis of the spectral density from the estimated acceleration data.

As mentioned already, the zenith wet delay residual is modeled with a random walk process so that the corresponding process noise covariance matrix can be expressed as following:

(11) $$Q_{\Delta ZWD}=q_{\Delta ZWD}\;\Delta t$$

It should be noted that the spectral density, $q_{\Delta ZWD}$ may be assigned with different values for reference stations and aircraft because the behavior of the zenith wet delay residuals are expected to be different from each other. The spectral density for zenith wet delay, $q_{\Delta ZWD}$ are determined by using the empirical auto-covariance functions method proposed by [4]. Then, 3 × 10^−10^ m^2^/Hz and 5 × 10^−8^ m^2^/Hz are chosen for the spectral densities for reference stations and aircraft, respectively. The process noise covariance matrix for DD ambiguities for L1 and wide-lane is modeled with zero matrix. The covariance matrix for DD ionospheric delay is provided by [3] as shown in Equation (12).

(12) $$Q_{ion}=\;\sigma_{\infty }^{2}\left(1-e^{-2\;\left(\left|\Delta t\right|/T+\left|\delta \right|/D\right)}\right)$$

where T = 74 min is correlation time, D = 1500 km is correlation distance; $\sigma_{\infty }^{2}=$2.0 m^2^ is variance.

### 2.2. GPS Measurement Equations

The DD GPS measurements are widely used for the relative positioning due to the fact that various systematic errors can be eliminated by differencing operation [16]. In other words, many error sources in the GPS measurements, for example, receiver and satellite clock errors, and atmospheric effects can be removed or significantly reduced in the DD measurements. However, the spatially correlated atmospheric errors should be modeled as the spatial separation between the two receivers increases. The Equation (13) shows the DD measurement equations for both L1 and L2 frequencies [17,18].

(13) $$\begin{array}{l} \Phi_{ij,1}^{kl}=\rho_{ij}^{kl}+T_{ij}^{kl}-\frac{I_{ij}^{kl}}{f_{1}^{2}}+\lambda_{1}N_{ij,1}^{kl}+\varepsilon_{ij,1}^{kl}\\ \Phi_{ij,2}^{kl}=\rho_{ij}^{kl}+T_{ij}^{kl}-\frac{I_{ij}^{kl}}{f_{2}^{2}}+\lambda_{2}N_{ij,2}^{kl}+\varepsilon_{ij,2}^{kl}\\ P_{ij,1}^{kl}=\rho_{ij}^{kl}+T_{ij}^{kl}+\frac{I_{ij}^{kl}}{f_{1}^{2}}+e_{ij,1}^{kl}\\ P_{ij,1}^{kl}=\rho_{ij}^{kl}+T_{ij}^{kl}+\frac{I_{ij}^{kl}}{f_{2}^{2}}+e_{ij,2}^{kl} \end{array}$$

where, subscript *i* and *j* indicate receivers while superscript k and ℓdenote satellites; ${\left(\cdot \right)}_{ij}^{k\ell }$ is double-differencing operator defined as ${\left(\cdot \right)}_{ij}^{k\ell }={\left(\cdot \right)}_{i}^{k}-{\left(\cdot \right)}_{j}^{k}-{\left(\cdot \right)}_{i}^{\ell }+{\left(\cdot \right)}_{j}^{\ell }$; $\Phi_{1}$, $\Phi_{2}$ are phase pseudo-range measurements on L1 and L2, respectively; $P_{1}$, $P_{2}$ are code pseudo-range measurements on L1 and L2, respectively; ρ is the geometric distance between the receiver and satellite; T is tropospheric delay, $f_{1}$ and $f_{2}$ are carrier frequencies of L1 and L2, respectively; $I/f_{1}^{2}$ and $I/f_{2}^{2}$ are ionospheric delays for L1 and L2, respectively; $\lambda_{1}$ and $\lambda_{2}$ are wavelengths of L1 and L2, respectively; $N_{1}$ and $N_{2}$ are ambiguities associated with L1 and L2, respectively. $\varepsilon_{1}$, $\varepsilon_{2}$, $e_{1}$, $e_{2}$ are measurement noises.

The DD GPS measurement equations shown in Equation (13) are nonlinear forms; thus, the linearization step is required. After linearization of Equation (13), the design matrix, H has the matrix form as shown in Equation (14).

(14) $$H=\left[\begin{array}{ccccccccccccccccc} h_{x_{i}} & h_{y_{i}} & h_{z_{i}} & h_{x_{j}} & 0 & 0 & h_{y_{j}} & 0 & 0 & h_{z_{j}} & 0 & 0 & h_{\Delta ZWD_{i}} & h_{\Delta ZWD_{j}} & \lambda_{1} & 0 & -1\\ h_{x_{i}} & h_{y_{i}} & h_{z_{i}} & h_{x_{j}} & 0 & 0 & h_{y_{j}} & 0 & 0 & h_{z_{j}} & 0 & 0 & h_{\Delta ZWD_{i}} & h_{\Delta ZWD_{j}} & \lambda_{2} & -\lambda_{2} & -f_{1}^{2}/f_{2}^{2}\\ h_{x_{i}} & h_{y_{i}} & h_{z_{i}} & h_{x_{j}} & 0 & 0 & h_{y_{j}} & 0 & 0 & h_{z_{j}} & 0 & 0 & h_{\Delta ZWD_{i}} & h_{\Delta ZWD_{j}} & 0 & 0 & 1\\ h_{x_{i}} & h_{y_{i}} & h_{z_{i}} & h_{x_{j}} & 0 & 0 & h_{y_{j}} & 0 & 0 & h_{z_{j}} & 0 & 0 & h_{\Delta ZWD_{i}} & h_{\Delta ZWD_{j}} & 0 & 0 & f_{1}^{2}/f_{2}^{2} \end{array}\right]$$

As shown in Equation (14), the components of the design matrix can be categorized into partial derivatives with respect to the coordinates for *i* (reference station) and *j* (aircraft), ZWD residual, DD ambiguities, and DD ionospheric delays.

Hence, the components corresponding to the coordinates of reference station and rover in the design matrix can be expressed as following:

(15) $$\begin{array}{ll} h_{x_{i}}=-\frac{x^{k}-x_{i,o}}{\rho_{i,0}^{k}}+\frac{x^{\ell }-x_{i,o}}{\rho_{i,0}^{\ell }}, & h_{x_{j}}=\frac{x^{k}-x_{j,o}}{\rho_{j,0}^{k}}-\frac{x^{\ell }-x_{j,o}}{\rho_{j,0}^{\ell }}\\ h_{y_{i}}=-\frac{y^{k}-y_{i,o}}{\rho_{i,0}^{k}}+\frac{y^{\ell }-x_{i,o}}{\rho_{i,0}^{\ell }}, & h_{y_{j}}=\frac{y^{k}-y_{j,o}}{\rho_{j,0}^{k}}-\frac{y^{\ell }-y_{j,o}}{\rho_{j,0}^{\ell }}\\ h_{z_{i}}=-\frac{z^{k}-z_{i,o}}{\rho_{i,0}^{k}}+\frac{z^{\ell }-z_{i,o}}{\rho_{i,0}^{\ell }}, & h_{z_{j}}=\frac{z^{k}-z_{j,o}}{\rho_{j,0}^{k}}-\frac{z^{\ell }-z_{j,o}}{\rho_{j,0}^{\ell }} \end{array}$$

The tropospheric delay consists of dry and wet components. Approximately 90% of the total zenith delay (TZD) is from the dry component, and about 10% is from the wet component [16]. It is well known that the dry component can be modeled with high accuracy while the wet component is less predictable. Therefore, the ZWD residual with respect to a priori value is estimated in this approach. Equation (16) shows the tropospheric delays between the receiver and satellite.

(16) $$\begin{array}{c} T_{i}^{k}=ZDD_{i}\;m_{i,D}^{k}+\left(ZWD_{i}+\Delta ZWD_{i}\right)\;m_{i,W}^{k}\\ T_{i}^{\ell }=ZDD_{i}\;m_{i,D}^{\ell }+\left(ZWD_{i}+\Delta ZWD_{i}\right)\;m_{i,W}^{\ell }\\ T_{j}^{k}=ZDD_{j}\;m_{j,D}^{k}+\left(ZWD_{j}+\Delta ZWD_{j}\right)\;m_{j,W}^{k}\\ T_{j}^{\ell }=ZDD_{j}\;m_{j,D}^{\ell }+\left(ZWD_{j}+\Delta ZWD_{j}\right)\;m_{j,W}^{\ell } \end{array}$$

where ZDD is modeled zenith dry delay; ZWD is modeled zenith wet delay; ΔZWD is ZWD residual; m is mapping function; subscript D and W indicate “dry” and “wet”, respectively.

The modeled ZDD, ZWD and their mapping functions are computed by using the Saastamoinen model [19]. The tropospheric delays in Equation (16) can be used to make the DD forms and the components of the design matrix correspond to the ΔZWD is as following:

(17) $$\begin{array}{l} h_{\Delta ZWD_{i}}=m\left(z_{i}^{k}\right)-m\left(z_{i}^{\ell }\right)\\ h_{\Delta ZWD_{j}}=-m\left(z_{j}^{k}\right)+m\left(z_{j}^{\ell }\right) \end{array}$$

To obtain the covariance matrix for the DD measurements, $R_{k}$, the error propagation law is applied to the covariance matrix for the un-difference (UD) measurements. The magnitudes of the noises for code and phase pseudo-range observations are set to decimetre-level and milimetre-level, respectively [14].

## 3. Results and Discussion

**Figure 1:**
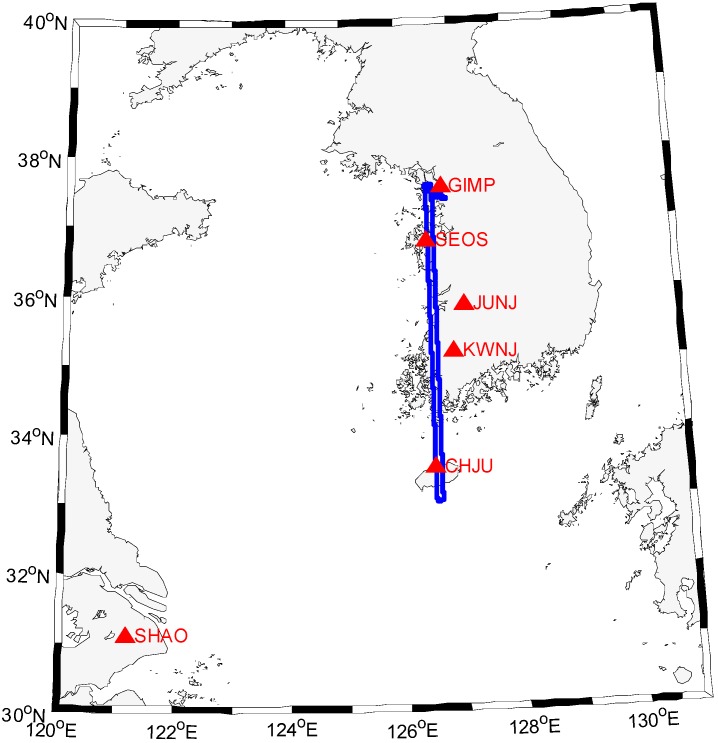
Ground track of aircraft and locations of CORS.

An airborne gravity survey was conducted in South Korea to develop a new geoid model in 2009. A Cessna Grand Caravan was used for the survey and it was flown with the help of autopilot at a constant altitude, *i.e.*, 10,000 feet, in order to get as smooth a flight as possible. The GPS data were collected from both the GPS receiver on board and six Continuously Operating Reference Station (CORS) with 1 s data interval. Figure 1 shows one of the trajectories of aircraft (blue line) and the locations of reference CORS used to demonstrate the proposed algorithm. The trajectories of the aircraft started and ended at the Gimpo (GIMP) airport and the total length of the aircraft trajectory is about 1200 km.

The coordinates of the reference stations in ITRF2005 frame are determined using the BERNESE GPS data processing software [20], and the results are shown in Table 1. The coordinates of reference stations are tightly constrained by assigning very small variance values (*i.e.*, 1 × 10^−10^ m^2^) to the initial covariance matrix.

For the medium to long baseline data processing scenario, the SHAO reference station is included in the data processing. Hence, the diameter of the CORS GPS network reaches up to approximately 900 km. The GPS data was collected on 11 January 2009 and the data span was 4 h and 18 min.

**Table 1:** ITRF2000 Cartesian coordinates for continuously operating reference station (CORS) (unit: m).

Station Name	X	Y	Z
GIMP	−3031,894.828	4054,598.988	3866,287.717
SEOS	−3042,060.369	4111,978.757	3797,578.729
JUNJ	−3124,886.919	4126,580.526	3714,170.141
KWNJ	−3134,404.485	4173,081.827	3654,100.961
CHJU	−3168,622.305	4277,489.565	3501,650.004
SHAO	−2831,733.444	4675,665.953	3275,369.395

**Figure 2:**
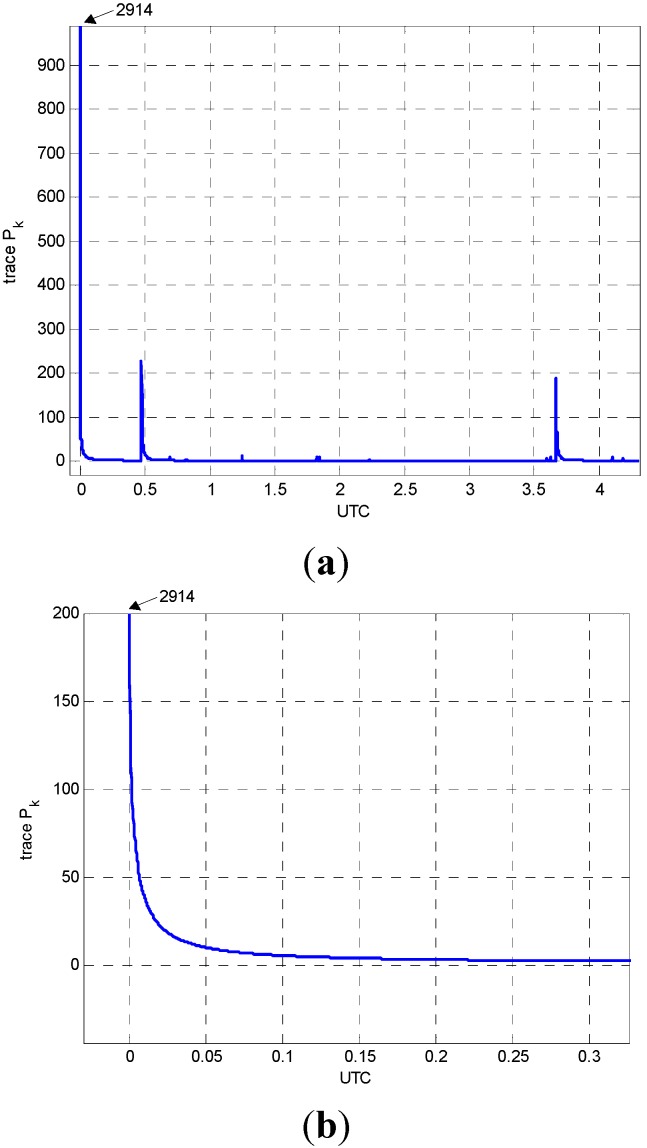
Convergence of the covariance matrix, P_k_: (**a**) whole trajectory; (**b**) beginning part of the trejectory.

As described in Section 2, the kinematic accelerations together with positions and velocities are estimated through the Kalman filter approach. The multi-baseline data processing is performed with radial type of network configuration. To evaluate the filter efficiency, the convergence of the covariance matrix P_k_ is examined. Figure 2a presents the variations of the trace of P_k_ with respect to time. As shown in Figure 2a, the trace of P_k_ converges rapidly with respect to time, which indicates the reliability of the filter. It is also notable that two peaks in Figure 2a correspond to the epochs at which new satellites are observed. Then, new states for DD ionospheric delays and ambiguities are included in state vector, and predefined variance values, *i.e.*, (0.5 m)^2^ for DD ionospheric delay and 1 × 10^10^ for DD ambiguity, are assigned to the new states.

**Figure 3:**
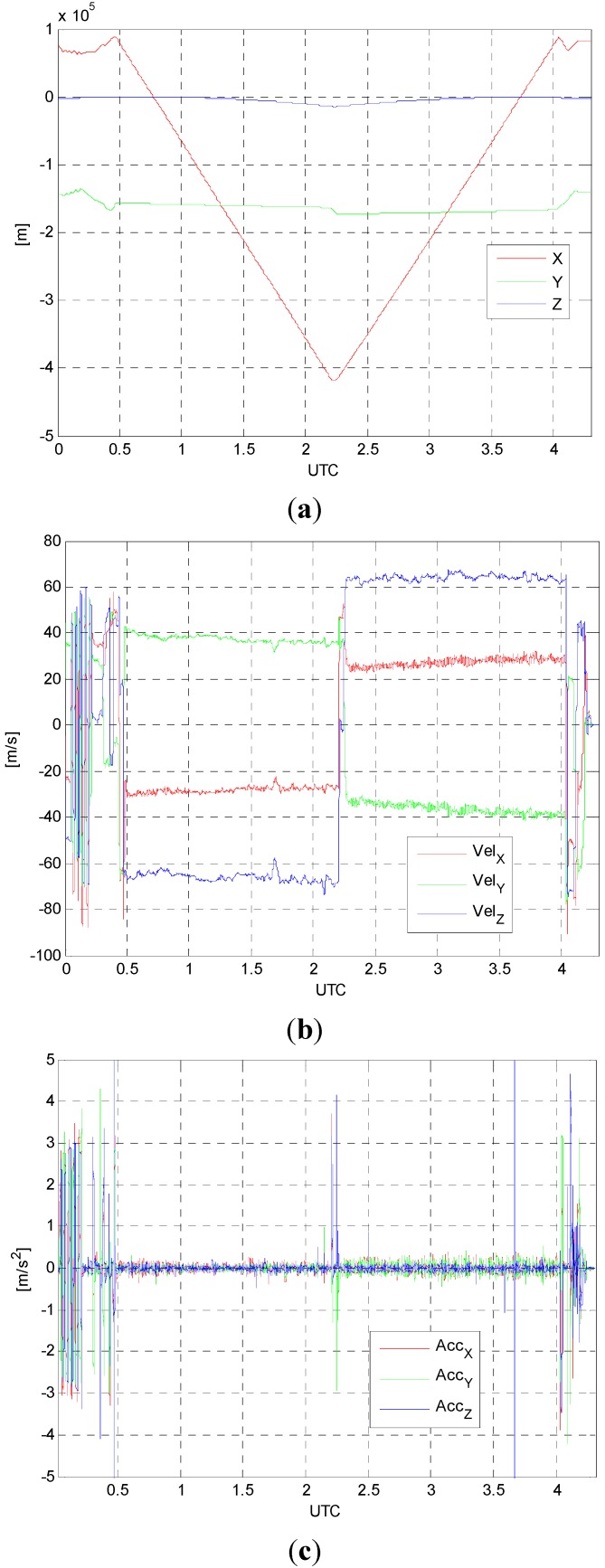
(**a**) Positions; (**b**) velocities; (**c**) accelerations.

Figure 3a–c present the estimated positions, velocities and accelerations of the aircraft with respect to time, respectively. The estimated states are the results from forward-backward smoothing.

As shown in Figure 3b, the constant velocities in most of the data span for airborne gravity survey are retained. The peaks observed in Figure 3c correspond to the aircraft maneuvering for takeoff, turnaround, and landing. Table 2 shows the statistics of the estimated accelerations which ranges from about −4.5 m/s^2^ to 4.5 m/s^2^.

**Table 2:** Statistical characteristics of estimated accelerations (unit: m/s^2^).

	$\ddot{x}$	$\ddot{y}$	$\ddot{z}$
Min.	−3.92	−4.18	−4.62
Max.	3.75	4.34	4.49
Std.	0.53	0.51	0.52

**Figure 4:**
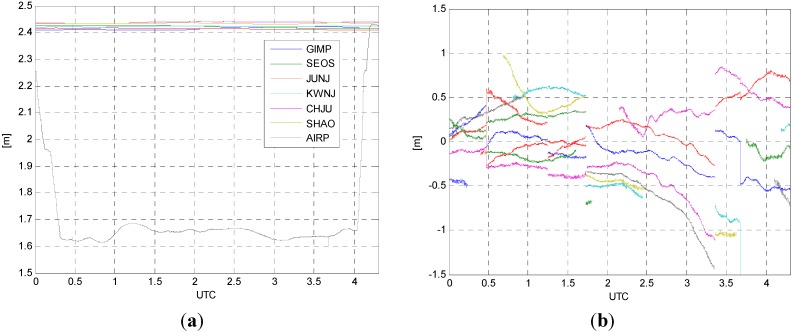
(**a**) Total zenith delays (TZDs); (**b**) DD ionospheric delays for SHAO-AIRP baseline.

The atmospheric effects are estimated in forms of ZWD residuals and DD ionospheric delays, respectively. As explained in Section 2, the ZWD residuals are estimated at each station including the aircraft and then final TZDs are computed by adding the modeled values to the estimated ZWD residuals. Figure 4a shows the final TZDs obtained from the data processing. As shown in Figure 4a, no significant variations of TZDs for the reference stations are observed while abrupt changes in the magnitude of values can be seen in the TZD estimated from the aircraft. This is caused by the fact that the modeled values are highly depended on the altitude of the aircraft. The DD ionospheric delays are also computed for each of the baseline and satellite pairs, and one example of the results for SHAO-AIRP baseline is shown in Figure 4b. Each continuous line corresponds to the DD ionospheric delay for each pair of GPS satellites. The variation of the DD ionospheric delays ranges from about −1 m to 1 m.

**Figure 5:**
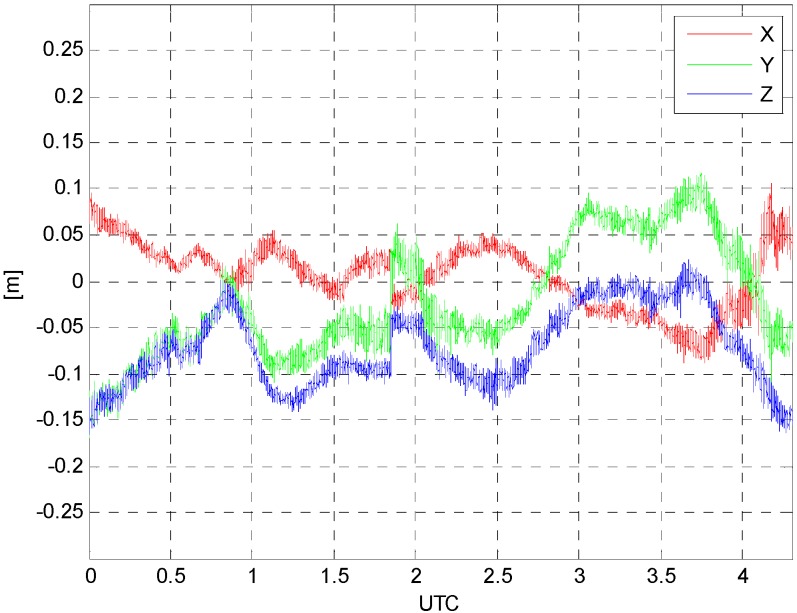
Differences between the estimated coordinates and reference values.

The next step is to evaluate the quality of the estimated kinematic accelerations once all the solutions are obtained through the proposed algorithm. However, there is a limitation on direct comparison because the reference values for kinematic accelerations are not available. Therefore, the coordinates of aircraft are computed using the same GPS datasets with the commercial software called Waypoint^®^, and the computed coordinates are used as reference values for the evaluation. Figure 5 presents the differences between the estimated coordinates using the proposed method and the reference values from the Waypoint software. The differences between the two solutions range from about −0.15 m to 0.1 m as shown in Table 3. From the results, one can conclude that the filter based on the proposed algorithm is working properly.

**Table 3:** Statistical characteristics of the differences between the estimated coordinates and the reference values (unit: m).

	Δx	Δy	Δz
Min.	−0.09	−0.17	−0.17
Max.	0.11	0.11	0.02
Std.	0.04	0.06	0.04

**Figure 6:**
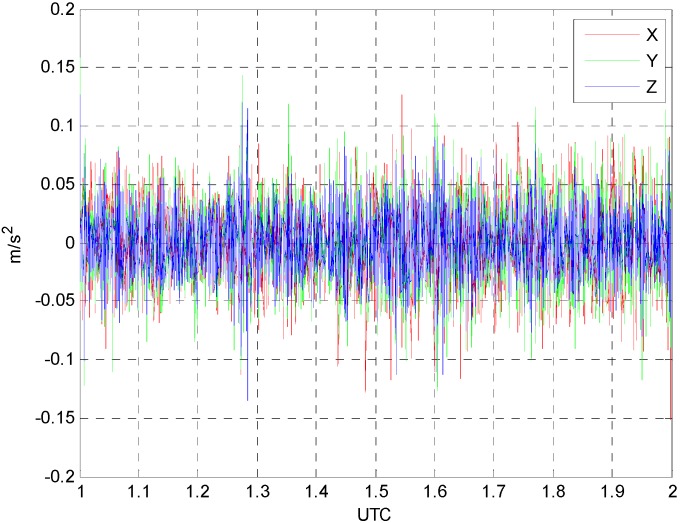
Differences between the estimated kinematic accelerations and reference values.

Then, the second-order time-derivatives of determined positions are applied to obtain the kinematic accelerations. It should be mentioned that taking the time-derivatives is performed after the data fitting with B-spline function. In the final step, the differences between the two kinematic accelerations are computed as shown in Figure 6. This procedure is applied to only 1 h data span, *i.e.*, 1–2 h (UTC) because no significant dynamics of the aircraft is observed. This also indicates that the actual gravity survey is performed during that time span.

Table 4 shows the statistical characteristics of the differences between the estimated kinematic accelerations and the reference values. The differences between the two solutions range from −0.15 m/s^2^ to 0.16 m/s^2^ and the standard deviation of the differences is about 0.03 m/s^2^. The mean value of the differences is 10^−5^ m/s^2^ level for each component.

From the results, one can state that the comparable kinematic accelerations for airborne gravity survey can be obtained by using the proposed algorithmIt should be mentioned that the kinematic acceleration for airborne gravimetry needs the accuracy at least at the level of 10^−4^ m/s^2^ to detect the gravity signal at the level of 10 mGal. In airborne gravimetry, however, a smoother is applied to the acceleration to extract meaningful gravity signal. Although the extraction of the gravity signal is out of the scope in this study, a B-spline smoother with window size of 120 s are applied to both accelerations and verified that the differences reside at the level of 10^−4^ m/s^2^. Therefore, one can conclude that the proposed algorithm is reliable for the application of kinematic accelerations. The validation for the proposed algorithm in this study, however, is performed with the dataset of one trajectory collected under the relatively low dynamic conditions. Therefore, it might be necessary to process more dataset under the high dynamic environments in the future.

**Table 4:** Statistical characteristics of the differences between the estimated kinematic accelerations and the reference values (unit: m/s^2^).

	$\Delta \ddot{x}$	$\Delta \ddot{y}$	$\Delta \ddot{z}$
Min.	−0.15	−0.13	−0.14
Max.	0.13	0.16	0.13
Std.	0.03	0.03	0.02

## 4. Conclusions

In this study, we proposed the kinematic GPS positioning algorithm using multiple reference stations for medium to large scale networks. The Kalman filter with PVA dynamic model is used for the kinematic positioning so that the kinematic acceleration information can be obtained simultaneously. For the long range kinematic applications, the tropospheric and ionospheric delays are modeled with random walk and first-order Gauss-Markov process models, respectively. The algorithm is implemented and tested with *in situ* airborne GPS data collected at 10,000 ft altitude with 1 s data interval. For the validation of the proposed algorithm, the positioning results are compared with the reference values first. The computed differences between the estimated and reference values range from −0.17 m to 0.11 m, which indicates the validity of the proposed algorithm. The estimated kinematic accelerations from the proposed algorithms are also compared with those from the second-order time-derivatives of the reference positions. The computed differences between the two solutions range from −0.15 m/s^2^ to 0.16 m/s^2^ and the standard deviation of the differences is about 0.03 m/s^2^. From these results, we can conclude that comparable kinematic accelerations, which can be used for airborne gravimetry, are obtainable using the proposed algorithm.
